# Impact of Peripheral Arterial Disease on Functional Limitation in Congestive Heart Failure: Results from the National Health and Nutrition Examination Survey (1999–2004)

**DOI:** 10.1155/2012/306852

**Published:** 2012-12-17

**Authors:** Bamidele A. Adesunloye, Ravinder Valadri, Nkechi M. Mbaezue, Anekwe E. Onwuanyi

**Affiliations:** ^1^National Institutes of Health (NIH), 9000 Rockville Pike, Bethesda, MD 20892, USA; ^2^Section of Cardiology, Department of Internal Medicine, Morehouse School of Medicine, Atlanta, GA 30310, USA; ^3^Department of Public Health, Rollins School of Public Health, Emory University School of Medicine, Atlanta, GA 30329, USA; ^4^Department of Internal Medicine, Morehouse School of Medicine, Atlanta, GA 30310, USA

## Abstract

*Background*. Peripheral arterial disease (PAD) often coexists with congestive heart failure (CHF) and can be masked by symptoms of CHF such as functional limitation (FL), a common manifestation for both. Therefore, we sought to estimate the prevalence of PAD and its independent association with FL in CHF. *Methods.* We conducted a cross-sectional study on National Health and Nutrition Examination Survey (NHANES) data from 1999 to 2004 to quantify weighted prevalence of CHF and PAD. Study cohort consisted of 7513, with ankle brachial index (ABI) measurements at baseline. Independent association of PAD (ABI ≤ 0.9) with FL in CHF was determined with multivariate logistic regression (MVLR). *Results.* Overall weighted PAD prevalence was 5.2%. CHF was present in 305 participants, and the weighted prevalence of PAD in this subgroup was 19.2%. When compared, participants with CHF and PAD were more likely to be older (*P* < 0.001), hypertensive (*P* = 0.005) and hypercholesterolemic (*P* = 0.013) than participants with CHF alone. MVLR showed that PAD (adjusted OR = 5.15; 95% CI: 2.2, 12.05: *P* < 0.05) and arthritis (adjusted OR = 2.36; 95% CI: 1.10, 5.06: *P* < 0.05) were independently associated with FL in CHF. *Conclusion.* Independent association of PAD with FL suggests the need for reinforced screening for PAD in individuals with CHF.

## 1. Introduction

 CHF continues to impose a major public health challenge, with a lifetime risk of 1 in 5, starting at age 40 years [1]. From an estimated 6.6 million heart failure patients in 2010, the prevalence is projected to increase by approximately 25% by the year 2030 [2]. FL is the hallmark of the CHF syndrome. It results from several factors including reduced tissue perfusion, impaired endothelial function [3], impaired calf muscle oxygen utilization from mitochondrial abnormalities [4], reduced oxidative enzyme capacity [5], and muscle atrophy [6, 7]. This dysfunctional state also applies to the respiratory muscles, resulting in reduced muscle strength, inefficient gas exchange, and contributing to poor functional performance in CHF [8, 9]. On the other hand, PAD patients have an arterial occlusive disease, in addition to the impairment of the microcirculatory mechanisms and calf muscle metabolism, similar to CHF. Although FL is a common manifestation of PAD and CHF, it is rarely attributed to PAD when both are present. The specific aims of the present study were (1) to determine the prevalence of PAD among noninstitutionalized US adults 40 years and older with CHF by using ABI measurements and (2) to examine the association between PAD and FL as measured by difficulty in walking among participants with CHF. 

## 2. Materials and Methods 

### 2.1. Study Population

The study population was derived from a nationally representative sample of United States population from the National Health and Nutrition Examination Survey (NHANES), 1999–2004. Noninstitutionalized persons were selected by using a stratified multistage sampling design by the National Center for Health Statistics. Low income persons, elderly, African Americans, and Mexican Americans were oversampled. Data files from interview, examination, and laboratory components were merged.

 Males and females aged ≥40 years that had their ABI measurements were included in the analysis. Exclusion criteria were individuals with missing data and an ABI >1.5, usually seen in people with noncompressible arteries due to medial arterial calcification [10, 11]. Predictor variable PAD was measured by hand-held Doppler probe method as established previously [12, 13] and defined as present when ABI <0.9 and absent when ABI ≥0.9, a cut-off value validated by Xu et al. [10]. Participants with CHF were separated out from the entire NHANES sample and categorized in two groups: those with PAD (CHF-PAD) and those without PAD (CHF). Outcome variable FL was recorded as a binary variable and prespecified as follows participants with FL at (1) quarter mile (2-3 blocks) distance, (2) 10 steps distance without resting, and (3) room-to-room distance on the horizontal level. HTN was recorded based on self-report, blood pressure ≥140/90 mmHg, or current use of medications for HTN. Hypercholesterolemia was recorded based on self-report, total cholesterol ≥240 mg/dL, or medication use for hypercholesterolemia. DM was recorded based on self-report and/or current medications use. Smoking was recorded based on self-report. Comorbid conditions recorded on the basis of self report were arthritis, CHF, emphysema, chronic bronchitis, and CAD. CHF diagnosis was based on Framingham CHF diagnostic criteria [14].

### 2.2. Statistical Analysis

 Continuous variables were summarized by mean and standard errors. Categorical variables were summarized by proportions. Differences in baseline characteristics between both groups were tested using Student's *t*-test for continuous variables and chi square test for categorical variables. MVLR analysis was used to test the independent association of PAD with FL. All tests were 2-tailed with alpha of 0.05. SPSS version 16 being used for the analysis. 

## 3. Results

**Figure 1:**
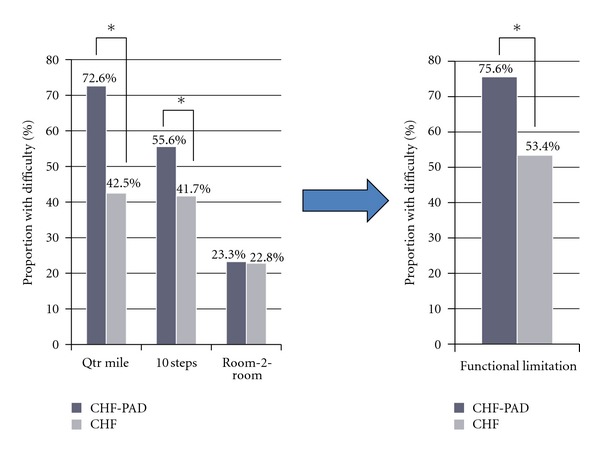
Distribution of functional limitation in CHF-PAD and CHF groups.

 A total of 7513 participants who had their ABI measured were screened for the presence of PAD and CHF. PAD was present in 591 (7.9%; weighted prevalence, 5.2%), while 305 (4.1%) participants had clinical diagnosis of CHF. PAD was present in 68 (22.2%; weighted prevalence, 19.2%) participants with CHF. Overall the cohort was predominantly white (79%), followed by non-Hispanic blacks (11.1%) and Mexican Americans (3.1%). The baseline characteristics of the CHF cohort are shown in Table 1. When compared with CHF group, CHF-PAD group was older, more likely to have HTN and hypercholesterolemia. FL was compared between CHF-PAD and CHF groups. When compared, CHF-PAD group had greater proportion of participants with at least one of the 3 prespecified FL criteria (75.6% versus 53.4%; *P* < 0.001) (Figure 1). However, in participants with the greatest FL, that is, symptomatic at room to room distance, there was no effect of PAD on functional performance. The proportion of participants with FL at quarter mile and 10 steps were similar (42.5% versus 41.7%) in the CHF group unlike in the CHF-PAD group, where more participants were symptomatic at quarter mile and 10 steps distances; 72.6% versus 55.6%, respectively, (Figure 1). PAD was independently associated with presence of FL in participants with CHF (OR = 2.7; CI: 1.33, 5.47; *P* < 0.05) (Table 2). The association remained significant (OR = 5.15; CI: 2.20, 12.05; *P* < 0.05) after adjusting for multiple covariates known to affect FL in CHF (Table 3). Covariates adjusted in the multivariate model included: age, gender, ethnicity, body mass index (BMI), and DM, HTN, hypercholesterolemia, smoking status, arthritis, emphysema, chronic bronchitis, and CAD. Apart from PAD, arthritis was also associated with FL on multivariate model (OR 2.36; CI: 1.10, 5.06; *P* < 0.05).

## 4. Discussion

 Our study estimated prevalence of PAD and its association with FL in noninstitutionalized individuals with CHF, in whom FL has been shown to be a valuable clinical parameter for risk stratification [15]. The cohort is from NHANES data, which is representative of non institutionalized adults in the United States. In this cohort, the overall weighted prevalence of PAD was 5.2% and 19.2% in the CHF subgroup. The analysis showed that PAD was independently associated with FL within the CHF cohort. The association was significant at univariate level with 2.7-fold greater likelihood of having FL, and the odds increased to 5.15, when adjusted for other confounding factors (Tables 2 and 3). Furthermore, presence of PAD was significantly associated with greater FL in individuals with CHF at all prespecified categories of FL, except for room-to-room distance. 

 Epidemiological studies estimating the prevalence of PAD were often done in heart failure naïve, otherwise, healthy individuals [16, 17]. Studies evaluating the prevalence of PAD in patients with CHF are sparse. One well-known study that estimated prevalence of PAD in chronic systolic heart failure which used data from Beta-Blocker Evaluation of Survival Trial (BEST) reported prevalence of PAD as 19.6% [18]. The same study also showed that the presence of PAD predicted a 40% increase in all-cause mortality, 63% increase in cardiovascular mortality, and 36% increase in all-cause hospitalization [18]. Apart from greater mortality, PAD has also been shown to independently predict incident HF. For example, Cardiovascular Health Study (CHS) reported a relative risk of 1.61 for incident HF in patients with baseline PAD [19]. However, these studies did not report on FL. Study by Hebert et al. [20] found similar overall PAD prevalence as our study and recommended the necessity to diagnose subclinical PAD in patients with CHF. However, disparity in the prevalence of PAD in racial sub-groups in our study can be attributed to the differences in the sample size of HF patients.

 The prevalence of PAD estimated from our entire study cohort is consistent with observations from prior studies, which ranged from 3% to 10%, using an ABI cut-off of <0.9, the most often used hemodynamic definition of PAD [16, 17]. We have used an ABI cut-off of <0.9 based on the AHA (American Heart Association) recommendations [21]. ABI test has been shown to be of great value because not only is it inexpensive and noninvasive [22] but also proved to be safe with a reasonably high sensitivity (90%) and specificity (98%) [23]. Nonetheless, there is considerable variation in the prevalence of PAD reported in the literature. This may be partly attributable to the differences in the population studied. For example, dramatic increase in the prevalence of PAD has been observed in patients older than 70 years [24]. In our study the mean age was 60.2 years, thus limiting the contribution of age on the reported PAD prevalence. However, amongst participants with CHF, the prevalence of PAD increased fourfold compared to the general population (Table 2). The observed increase in the prevalence of PAD in CHF is not only consistent with prior observation from the BEST study [18], but may also reflect the fact that CHF and PAD share common risk factors [18, 24]. 

**Figure 2:**
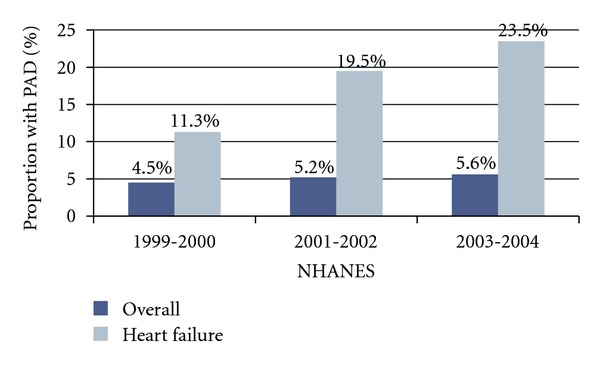
Weighted prevalence of PAD for each survey period.

 Prior studies from NHANES data suggest that PAD defined by ABI of ≤0.9 was more common in non-Hispanic AAs (7.8%) followed by Whites (4.4%). This association is further supported by study (GENOA) Genetic Epidemiology Network of Arteriopathy [25] in which the racial difference was not explained by difference in other conventional risk factors for atherosclerosis. Both the prevalence and the incidence of PAD have been shown to increase in direct proportion with increasing age [26]. Smoking has a direct causal relationship with PAD as shown by Fowkes et al. [27]. Likewise, other risk factors for PAD supported by evidence are DM [28, 29], HTN, and hypercholesterolemia [30]. These same risk factors are well established in the causation and progression of CHF. As we found in this study, there was significant increase in occurrence of key risk factors in the CHF-PAD group compared to CHF group (Table 1). Selvin and Erlinger reported the weighted prevalence of PAD in NHANES survey period 1999-2000 as 4.3% [26]. This is similar to the weighted prevalence of 5.2% reported in our study which spanned the survey periods from 1999 to 2004. However, as shown in Figure 2, there were differences in the prevalence of PAD in individuals with CHF. Prevalence of PAD increased in each successive NHANES survey period from 1999 to 2004. Further examination of the data from these three survey periods showed that there were a greater percentage of individuals with HTN, hypercholesterolemia, and DM in 2003-2004 NHANES compared to the others. In addition, there were more AAs surveyed in 2003-2004 period TABLE (Figure 2 and Table 4).

 FL has been shown to guide both medical as well as device management in patients with CHF, and it is an important indicator of prognosis. As far as we know, whether the presence of PAD undermines the value of FL as a prognostic tool has not been tested. We do not have data on functional limitation specifically measured in typical New York Heart Association (NYHA) functional classification system, which is a widely practiced method of ascertaining FL in patients with CHF [15]. However, the prespecified definitions for FL closely correspond to the crude definitions used in clinical practice, where FL at quarter mile distance is generally considered as NYHA class II (mild symptoms and slight limitation during ordinary activity), FL at 10 steps distance is considered as NYHA class III (marked limitation in activity due to symptoms, even during less-than ordinary activity), and FL at room to room distance is considered as NYHA class IV (experience symptoms even while at rest) [15]. Evidence from randomized controlled trials (RCTs) mandates escalation of conventional heart failure therapy in direct proportion with FL. Medical therapy in CHF escalates starting with angiotensin converting enzyme inhibitors [31], and Beta Blockers [32, 33] at NYHA class I and II, to Aldactone [34, 35], bidil [36], and inotropic therapy [37] as the FL deteriorates to NYHA class III and IV. Similarly, device therapy in CHF escalates to cardiac resynchronization therapy (CRT) when FL deteriorates to NYHA class III in appropriate clinical settings [38], and left ventricular assist devices (LVAD) [39] come on board as a bridge to cardiac transplantation as FL worsens to NYHA class IV. Therefore, it is of paramount importance to thoroughly screen for PAD and other factors that can impact functional status. Although the current risk stratification algorithms do account for various established cardiovascular risk factors that may influence outcomes in CHF [40], PAD is conspicuously absent. 

 CHF results in alteration of skeletal and respiratory muscle function which in turn worsens the CHF syndrome [11, 14–19, 21, 23, 24]. It has been shown that with appropriate exercise training, some of these dysfunctional elements can be reversed such as the increase in the skeletal muscle mitochondria ATP production as shown by Williams et al. [41]. However, in patients with PAD and CHF, claudicating calf pain from PAD limits their ability to exercise and potentially preclude them from achieving maximal beneficiary effects of exercise training. Moreover, as reported by Fowkes et al. patients with PAD are often asymptomatic [27], but even when symptoms are present, it may be extremely difficult for some CHF patients to discern claudicating symptoms from fatigue due to chronic low output state or poor effort tolerance. Therefore, PAD can often be missed in the risk stratification of patients with CHF and FL. 

 Our study provides the evidence for independent association of PAD with FL in CHF. These data highlight that PAD is masked in CHF patients and reinforces the need to maintain a high index of suspicion for PAD. Our study results in terms of independent association of PAD with FL are consistent with the results from the study by Jones et al. [42] which also showed independent association of PAD with poor exercise capacity and poor response to exercise training. However we do not have mortality data to verify the predictive value of PAD in HF as observed in the study by Jones et al. [42]. 

 Our analysis should be interpreted in the context of several limitations. Our study is retrospective and the data is cross-sectional, limiting us from commenting on any causal relationship between PAD and FL in participants with CHF. Although ABI has been shown to be a sensitive and specific clinical tool for PAD, it was primarily derived from patients without CHF [43]. Therefore its validity in CHF patients is uncertain. Moreover, these patients are more likely to be older have DM and HTN, conditions associated with vascular sclerosis and hardening of the arterial walls resulting in elevated ABI estimates and underestimation of PAD [44, 45]. However, we excluded participants with ABI values greater than 1.5. Additionally, because relatively lower ABI scores are generally reported in AA compared to non-Hispanic Whites, there may have been an overestimation of prevalence of PAD in AA. Furthermore, ABI has limitations as diagnostic test for PAD but can be effective as a screening tool. However, this study was not designed to test this question. Other areas of concern include the accuracy of reporting FL and the relatively small sample size of patients with CHF, although derived from a nationally representative cohort. Finally, we have not observed an association between PAD and established risk factors for PAD such as smoking, DM, and CAD. This deviation can be explained by the fact that measured associations in our study are against patients with CHF, who most often share the same risk factors.

## 5. Conclusions

 This study showed that participants with CHF were over 3 times more likely to have PAD compared to the general population and that the odds of FL were 5 times greater in those with PAD after adjusting for confounders. We propose that a protocol to screen for PAD should be an important component of a CHF disease management program.

## Figures and Tables

**Table 1:** Baseline characteristics (CHF patients only).

Characteristic	CHF-PAD (*n* = 68)	CHF (*n* = 237)	*P* value
Age, mean (SE)	72.8 (1.2)	64.1 (1.0)	<0.001*
BMI, mean (SE)	29.8 (1.1)	29.4 (0.4)	0.368
Male, %	58.6	61.6	0.715
Race, %			0.845
Non-Hispanic White	78.7	79.4	
Non-Hispanic Black	12.6	9.6	
Mexican American	2.3	3.8	
Diabetes, %	35.3	23.0	0.105
Hypertension, %	87.3	69.5	0.005*
Hypercholesterolemia, %	76.6	56.3	0.013*
Smoker, %	80.3	68.9	0.141
Arthritis	62.1	63.4	0.855
Emphysema	19.5	13.4	0.286
Chronic bronchitis	23.9	18.9	0.446
Coronary artery disease	44.9	52.9	0.287

*Denotes *P* < 0.05.

BMI: body mass index; SE: standard error; CHF: congestive heart failure; PAD: peripheral arterial disease.

**Table 2:** Simple logistic regression analysis showing association of each variable and functional limitation in patients with CHF.

Factor	Unadjusted OR	95% CI
PAD	2.70	1.33–5.47*
Age	0.79	0.32–1.93
Male	0.50	0.35–1.03
Ethnicity		
Non-Hispanic Black	2.10	0.73–6.03
Mexican American	0.30	0.13–0.67
BMI	0.98	0.50–1.95
Diabetes	0.93	0.50–1.85
Hypertension	1.34	0.61–2.93
Hypercholesterolemia	1.55	0.82–2.91
Smoking	0.76	0.32–1.79
Arthritis	2.74	1.56–4.80*
Emphysema	1.22	0.39–3.85
Chronic bronchitis	1.65	0.54–5.05
Coronary artery disease	0.99	0.52–1.90

*Denotes *P* < 0.05. OR: odds ratio; CI: confidence interval; CHF: congestive heart failure; PAD: peripheral arterial disease.

**Table 3:** Multivariate logistic regression analysis showing association between PAD and functional limitation in patients with CHF.

Factor	Adjusted OR	95% CI
PAD	5.15	2.20–12.05*
Age	0.43	0.15–1.20
Male	0.47	0.20–1.11
Ethnicity		
Non-Hispanic Black	1.97	0.69–5.68
Mexican American	0.24	0.09–0.69
BMI	0.63	0.26–1.51
Diabetes	0.58	0.21–1.60
Hypertension	1.13	0.49–2.58
Hypercholesterolemia	0.86	0.42–1.79
Smoking	0.56	0.18–1.76
Arthritis	2.36	1.10–5.06*
Emphysema	1.07	0.25–4.51
Chronic bronchitis	1.24	0.32–4.90
Coronary artery disease	1.26	0.61–2.60

*Denotes *P* < 0.05. OR: odds ratio; CI: confidence interval; CHF: congestive heart failure; PAD: peripheral arterial disease.

**Table 4:** Weighted prevalence of risk factors from 1999 to 2004 in the NHANES data.

Characteristics	1999-2000	2001-2002	2003-2004
Ages, mean (SE)	56.4 (0.3)	55.5 (0.4)	56.3 (0.4)
Non-Hispanic Blacks, %	8.9	9.0 ↑	9.8 ↑↑
Diabetes, %	8.6	9.1 ↑	10.7 ↑↑
Hypertension, %	47.1	44.7	50.2 ↑
Hypercholesterolemia, %	45.8	43.8	49.6 ↑
Smokers, %	57.4	54.8	57.2
CHF, %	2.9	2.6	3.2

CHF: congestive heart failure; PAD: peripheral arterial disease.

## Abbreviations

CHF: Congestive heart failure
PAD: Peripheral arterial disease
FL: Functional limitation
DM: Diabetes mellitus
HTN: Hypertension
CAD: Coronary artery disease
AA: African american.
